# Differences in Simulated Doctor and Patient Medical Decision Making: A Construal Level Perspective

**DOI:** 10.1371/journal.pone.0079181

**Published:** 2013-11-14

**Authors:** Jiaxi Peng, Fei He, Yan Zhang, Quanhui Liu, Danmin Miao, Wei Xiao

**Affiliations:** 1 Department of Psychology, Fourth Military Medical University, Xi’an, China; 2 School of Public Management, Northwest University, Xi’an, China; University of Leicester, United Kingdom

## Abstract

**Background:**

Patients are often confronted with diverse medical decisions. Often lacking relevant medical knowledge, patients fail to independently make medical decisions and instead generally rely on the advice of doctors.

**Objective:**

This study investigated the characteristics of and differences in doctor–patient medical decision making on the basis of construal level theory.

**Methods:**

A total of 420 undergraduates majoring in clinical medicine were randomly assigned to six groups. Their decisions to opt for radiotherapy and surgery were investigated, with the choices described in a positive/neutral/negative frame × decision making for self/others.

**Results:**

Compared with participants giving medical advice to patients, participants deciding for themselves were more likely to select radiotherapy (F_1, 404_ = 13.92, p = 011). Participants from positive or neutral frames exhibited a higher tendency to choose surgery than did those from negative frames (F_2, 404_ = 22.53, p<.001). The effect of framing on independent decision making was nonsignificant (F_2, 404_ = 1.07, p = 35); however the effect of framing on the provision of advice to patients was significant (F_2, 404_ = 12.95, p<.001). The effect of construal level was significant in the positive frame (F_1, 404_ = 8.06, p = 005) and marginally significant in the neutral frame (F_2, 404_ = 3.31, p = 07) but nonsignificant in the negative frame (F_2, 404_ = .29, p = 59).

**Conclusion:**

Both social distance and framing depiction significantly affected medical decision making and exhibited a significant interaction. Differences in medical decision making between doctors and patients need further investigation.

## Introduction

People encounter situations requiring decision making; however, only some decisions are formed independently [1], [2]. In many cases, people approach friends or specialists for advice and occasionally advise others. The final decision is usually a combination of the opinion of an individual and that of the adviser. In the medical field, patients are confronted with diverse medical decisions, such as which medicine to take or whether to undergo surgical treatment. Often lacking relevant medical knowledge, patients refuse to independently decide and instead generally rely on doctors for advice. Studies have shown that medical advice exerts the most significant influence on the medical decisions of patients [3]. However, differences in perspectives between doctors and patients as they make medical decisions need to be determined, especially in relation to doctors providing advice to others and patients deciding for themselves. Research conducted on this topic is currently limited. Marteau (1989) initially explored this area, with focus on the different effects of framing information on the decisions of doctors and patients at different risk levels, rather than the characteristics of independently formulated medical decisions and those based on other opinions [4]. The present research investigated the characteristics and differences in doctor–patient medical decision making on the basis of construal level theory (CLT).

### 1.1 Construal Level Theory

Construal level theory is a cognitive-oriented social psychological theory that has rapidly developed in recent years [5]. The theory states that people cognitively represent objects at different levels of abstraction called construal levels (CLs), which are determined by the psychological distance between a subject and the object of cognition [6], [7]. Objects that are psychologically distant from the self are associated with a high CL that represents events in terms of abstract and schematic concepts; such a CL focuses on core and integrated features. Objects that are psychologically proximate are associated with a low CL; such a CL focuses on the periphery and the detailed local features [8]–[11]. Metaphorically, an individual operating at a high CL is similar to a bird flying over a forest and viewing an entire landscape, whereas a person operating at a low CL is akin to a small creature on the forest floor observing individual trees and leaves.

Four main types of psychological distance affect CL: temporal distance (present vs. future), spatial distance (near vs. far), social distance (self vs. others), and probability (events of a high probability vs. events of a low probability). The farther the temporal, spatial, and social distance and the lower the subjective probability for the event to occur, the greater the tendency for people to employ a high CL rather than a low one [7]–[10]. An example is in considering the influence of social distance on CL in deciding which job offer to accept. In deciding for oneself (proximal social distance), an individual may view the situation at a low proximal distance and focus on concrete and detailed features, such as pay, work hours, and work location. In deciding for others (distant social distance), the individual may view the situation from a high CL, and the emphasis would be on the most important core features, such as the sense of self-satisfaction that the position can provide [12].

Nan stated that according to CLT, differences in mental representations of events associated with large or small psychological distance can have significant evaluative consequences; i.e., a low CL should be more influential when psychological distance is small than when it is large, and vice versa [13]. In the study by Ebert, for example, the participants were asked to list the long-term benefits (i.e., high-level construals) and the short-term costs (i.e., low-level construals) of a particular course of action. Half of the participants rated the importance of long-term benefits and short-term costs for themselves, whereas the other half assessed the rating for their friends. Ebert found that short-term costs were rated as much more important than long-term benefits, especially when the assessment is initially conducted for oneself and not among friends. Meanwhile, long-term benefits are perceived as equally important under the two conditions [14]–[16].

### 1.2 Framing Effects in Medical Situations

CLT is not the only cognitive theory that offers insights into medical decision making. Considerable research demonstrates that framing effects can influence medical decisions as well. The framing effect refers to a situation in which the same problem is presented using different representations of information (or frames); people apply significant changes to their decisions or even reverse their decisions [17].

A number of studies reported that the framing effect stably exists in medical situations. For example, when Bigman, Cappella, and Hornik described the effect of a human papilloma virus (HPV) prophylactic vaccines to some participants as 70% effective (positive frame) and to others as 30% ineffective (negative frame), different results were generated [18]. Despite the similarity of information contained in both frames, the participants who received the positive frame perceived the HPV vaccine to exhibit a sufficient prophylactic effect and were thus more willing than those who received the negative frame to receive the vaccine. In another study, Gerend and Cullen informed a group of participants about the benefits of alcohol abstinence and another group about the harm of alcohol abuse; the authors found a better dissuasive effect when a positive frame was used than when a negative frame was used [19]. By contrast, Maheswara and Meyers-Levy reported that compared with a positive frame, a negative frame is more effective in advising people to engage in blood cholesterol screening [20] and encouraging individuals to perform breast self-examination [21].

To reconcile the different findings, Retamero and Galesic suggested that gain frames can more effectively promote disease prevention behaviors, whereas loss frames can more effectively encourage disease detection behaviors [22]. Framing may also influence the choice of medical treatment. Armstrong et al. randomly assigned participants to one of three groups; each group was presented with information about two treatment programs. The first group was presented with mortality curves to describe the treatments, whereas the second group was shown survival curves. The third group was shown both survival and mortality curves. The results indicated that participants who were shown the mortality curves were significantly less likely to prefer preventive surgery than were those from the other two groups [23].

Conflict between doctors and patients has become severe in China [24]. More than 10000 medical personnel yearly are reportedly harassed by patients and their relatives in China. According to the Ministry of Health, cases of medical violence reached 10248 in 2006 and increased to 17234 in 2010 [25]. These incidents are attributed to the imperfect medical system and poor communication between doctors and patients. The Chinese medical system employs the paternalistic medical decision-making model, in which doctors have the right to decide on patient medication, as well as the treatment or procedure, without necessarily discussing these decisions with the patient [26]. Thus, differences in decision-making between doctors and patients in the Chinese context must be explored.

The purpose of the current research was to investigate two proposed variables, CL and framing, that affect medical decision-making by using the same design. First, we manipulated the social psychological distance variable (self-decision vs. giving advice to another). We predicted that social psychological distance would lead to differences in employed CL and subsequently affect decision-making such that the results are consistent with the findings in previous studies on CL effects. We then manipulated the second variable, i.e., the process by which medical decision results are framed. We predicted that regardless of positive, negative, or neutral framing, medical decisions would be affected. Finally, this study investigated the effect of the interaction between CLs and frame descriptions on medical decision-making.

## Methods

### 2.1 Participants

The participants in this study consisted of 420 first-year undergraduates from a medical university in China. These participants specialized in clinical medicine and participated for extra course credits and a ballpen as a gift. The reasons these participants were recruited were as follows: (1) We assumed that differences in doctor–patient decision making are caused primarily by differences in medical knowledge and CLs between doctors and patients; thus, the recruitment of university students controls the influence of medical knowledge. (2) The selected participants were at the initial stage of their training; thus, their medical knowledge is comparable to that of patients who mostly have basic knowledge about diseases. In addition, the selected participants were at the preparatory stage of becoming doctors and thus eager to be involved. The 420 medical students were asked to make medical decisions as clinical doctors or cancer patients. We distributed 420 inventories of which 404 were identified as valid, thus obtaining a recovery rate of 96.19%. Among the valid inventories, 91 were from female participants (22.5%), and 313 were from male participants (77.5%).

All participants provided their written informed consent before completing the measures. The research described in this paper meets the ethical guidelines of the Fourth Military Medical University and has been approved by the ethics committee of Fourth Military Medical University. Moreover, the research was conducted in adherence to the legal requirements of the People’s Republic of China.

### 2.2 Materials

We adopted the research material developed for a classic medical decision-making problem from the Adult Decision-Making Competence Inventory [27]. We adjusted the survival rate based on our previous study. The medical decision-making problem is detailed in the following scenario. A decision maker must decide between surgery or radiation therapy. Surgical treatment exhibits a lower treatment survival rate (50%) but a relatively higher five-year survival rate (40%), whereas radiation therapy has a higher treatment survival rate (100%) but a lower five-year survival rate (20%). We described the outcome of these two treatment programs in three frames: (a) lives saved, (b) lives lost, and (c) lives saved and lost. We also varied the social distance by setting the problem of participants as either (a) deciding for themselves or (b) providing advice to a patient (Table 1).

**Table 1:** Research materials.

	Making Decision for Oneself	Giving Advice for Patients
**Positive**	Your doctor tells you that you have cancer requiring treatment. Yourchoices are as follows: Surgery. Among 100 patients who undergosurgery, 50 live through the operation, and 40 remain alive at the endof five years. Radiation therapy. Among 100 patients undergoingradiation therapy, all live through the treatment, and 20 remain aliveat the end of five years. Which treatment would you choose?	One of your patients was diagnosed with cancer requiring treatment. The patient’s choices are as followsSurgery. Among 100 patients who undergo surgery, 50 live through the operation, and 40 remain alive at the end of five years. Radiation therapy. Among 100 people who undergo radiation therapy, all live through the treatment, and 20 remain alive at the end of five years. Which treatment would you advise your patient to choose?
**Negative**	Your doctor tells you that you have cancer requiring treatment. Yourchoices are as follows: Surgery. Among 100 people who undergosurgery, 50 die from the operation and 10 of the 50 survivors die by theend of five years. Radiation therapy. Among 100 patients undergoingradiation therapy, none die during the treatment, and 80 die by the endof five years. Which treatment would you choose?	One of your patients was diagnosed with cancer requiring treatment. The patient’s choices are as follows: Surgery. Among 100 people who undergo surgery, 50 die from the operation and 10 of the 50 survivors die by the end of five years. Radiation therapy. Among 100 people who undergo radiation therapy, none die during the treatment, and 80 die by the end of five years. Which treatment would you advise your patient to choose?
**Neutral**	Your doctor tells you that you have cancer requiring treatment. Yourchoices are as follows: Surgery. Among 100 patients who undergosurgery, 50 live through the operation, and 50 die from the operation.At the end of five years, 10 of the 50 survivors are dead, and 40 remainalive. Radiation therapy. Among 100 patients who undergo radiationtherapy, all live through the treatment. At the end of five years, 80 aredead and 20 remain alive. Which treatment would you choose?	Imagine that one of your patients was diagnosed with cancer requiring treatment. The patient’s choices are as follows: Surgery. Among 100 people who undergo surgery, 50 live through the operation, and 50 die from the operation. At the end of five years, 10 of the 50 survivors are dead and 40 are alive. Radiation therapy. Among 100 patients who undergo radiation therapy, all live through the treatment. At the end of five years, 80 are dead and 20 remain alive. Which treatment would you advise your patient to choose?

### 2.3 Procedure

We randomly assigned participants to one of six groups on the basis of a grid of two CLs (self or others) in three frames (positive, negative, and neutral) using a between-subject design. We used proportionate random sampling to control for gender. Responses to the decision-making problem were ranked on a 6-point Likert scale, ranging from 1 (confidently selecting the radiation therapy program) to 6 (confidently selecting the surgery program). While using a simple dichotomous scale where a participant chooses one of the two treatments, he/she also uses a 6-point scale to favor one procedure over the other because no mid-point exists. In addition, the 6-point scale allowed us to determine the strength of the choice made by the participants [24]. The participants were informed that they were participating in a psychological investigation in which no answer was considered right or wrong. CL and frame were identified as the two independent variables, whereas the responses of the participants to the decision-making problem comprised the dependent variable. Data analyses were performed using SPSS 16.0.

## Results

To verify that the proportion of males to females was the same in all six conditions, we conducted a chi-squared test and found no significant difference (χ^2^_5, 404_ = 1.85, p = .87). The number of males and females under each condition is listed in Table 2. Using one of the sample t-tests, we tested the mean decision score in each cell (see Table 2 for means) against a score of 3.5. The t-tests demonstrated that in general, the participants exhibited a tendency to select radiation therapy. However, no significant tendency to give advice to others was observed in a positive frame (t_65, 66_ = −.47, p = .64).

**Table 2:** Sex Structure and Decision Making by Social Distance and Framing.

Construal Level	Frame	Male N (%)	Female N (%)	Decision (M±SD)	*t*
**Low**	Positive	51(79.7%)	13(20.3%)	2.64±1.52	−4.54***
**Low**	Neutral	50(73.5%)	18(26.5%)	2.56±1.68	−4.73***
**Low**	Negative	51(75.0%)	17(25.0%)	2.28±1.31	−7.66***
**High**	Positive	16(24.2%)	50(75.8%)	3.41±1.57	−47
**High**	Neutral	15(20.0%)	60(80.0%)	3.03±1.43	−2.86**
**High**	Negative	12(19.0%)	51(81.0%)	2.16±1.23	−8.45***

We further tested the effects of social distance and frame description by 2×3 ANOVA. The results indicate that the main effect of social distance was significant (F_1, 404_ = 13.92, p = .011). The participants demonstrated a higher tendency to select radiation therapy when making clinical decisions for themselves than when giving advice to patients (mean difference (MD) equals.37). The main effect of framing was also significant (F_2, 404_ = 22.53, p<.001). LSD post-tests indicate that participants who were exposed to the mortality frame showed a stronger preference for low-risk treatment in the form of radiation therapy than did participants who were exposed to the survival (MD = .81, p<.001) and neutral (MD = .58, p = .001) frames. No significant difference was indicated in the decisions made by participants who were exposed to the positive and neutral frames (MD = .18, p = .20).

**Figure 1:**
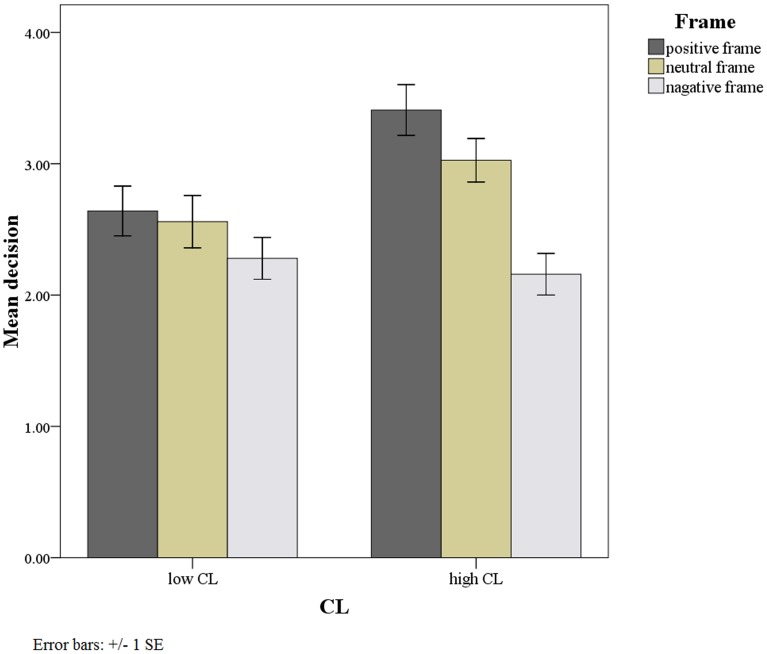
Framing Effects within Social Distance.

Social distance and framing (F_1, 404_ = 6.69, p = .045) exhibited no significant interaction. A simple effects analysis of the framing factor in each social distance reveals that the framing effect was nonsignificant at low social distance (F_2, 404_ = 1.07, p = .35), but significant at high social distance (F_2, 404_ = 12.95, p<.001; Figure 1). We also performed an LSD post-test and found that at high social distance, the participants who were exposed to a negative frame preferred radiation therapy more than did those who were exposed to a positive (MD = 1.25, p<.001) or a neutral (MD = .87, p = .001) frame. No significant difference was indicated in the decision made between the positive and the neutral frames at high or low social distances (MD = .24, p = .11).

**Figure 2:**
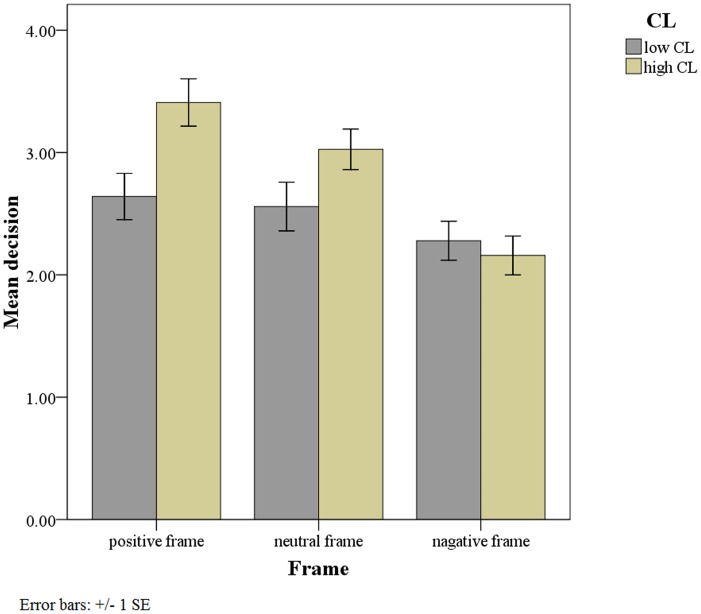
Social Distance Effects within Frames.

In a separate analysis, the simple effects of the construal were examined in each frame. The effect of social distance on medical decision-making varied across different frames. The effect was significant in the positive frame (F_1, 404_ = 8.06, p = .005) and marginally significant in the neutral frame (F_2, 404_ = 3.31, p = .07). Similar tendencies were demonstrated in these two frames. Compared with giving advice to patients, making decisions for oneself exhibited a greater preference for radiation therapy. Social distance exerted no significant influence on medical decision-making in the negative frame (F_2, 404_ = .29, p = .59; Figure 2).

## Discussion

### 4.1 Main Effect of Social Distance

In general, the participants preferred radiation therapy to surgery; this finding reveals the effect of time without people focusing on the gains or losses of the present than those of the future [28], [29]. For example, despite their awareness of the dangers of drug abuse, drug addicts still abandon long-term interests (e. g., health, family, social relations) in exchange for instant pleasure. In the current study, the majority of the participants focused on the treatment survival rate rather than the five-year survival rate because of time discounting. In addition, the preference for radiation therapy over surgery may be attributed to the “certainty effect” introduced by Tversky and Kahneman [17]. This effect pertains to the overweighing of outcomes obtained with certainty relative to those that are merely probable. From this perspective, the participants should also prefer the treatment that ensures a certain survival level with reduced longevity (i.e., radiation therapy) to that which provides probable survival with increased longevity.

We also found a significant main effect of social distance. Compared with participants giving advice to patients, participants independently making their decisions were more likely to choose radiation therapy treatment, which showed a high treatment survival rate but a relatively low five-year survival rate. From the perspective of CLT, making decisions for oneself has a low CL, resulting in people focusing on the present; meanwhile, giving advice to others employs a high CL, resulting in people focusing on the future [6]–[8]. In the present study, radiotherapy obtained a better immediate outcome but a worse five-year outcome, compared with surgery. A low CL involved in independent decision making rather than in giving advice to patients can explain why the participants preferred radiotherapy to surgery. Another explanation may be the tendency to focus on the desirability of a high CL and the feasibility of a low CL. Previous reports indicate that people attach importance to the absolute desirability of a benefit when participating at a high CL and consider feasibility at a low CL. 16 If long-term survival is assumed to be the goal of treatment in the current study, then radiation therapy with its low five-year survival rate would have a lower desirability than surgery. The high treatment survival rate of radiation therapy indicates that compared with no treatment, radiation therapy is highly feasible and can more likely achieve an increased five-year survival rate. By contrast, surgery exhibited a low treatment survival rate (low feasibility) but a high five-year survival rate (high desirability). Thus, people tend to select radiation therapy when deciding for themselves because a low CL directs them to focus on the higher feasibility of radiation therapy and overlook the higher desirability of surgery. The reverse tendency explains why the participants giving advice to patients preferredthe surgery program; the high CL directs them to focus on the higher desirability of surgery and overlook the higher feasibility of radiation therapy.

### 4.2 Framing Effect in Medical Situations

The participants who were presented with either the survival probabilities or both survival and mortality probabilities of the treatment options were more inclined to choose the surgery treatment, compared with those who were presented with only the mortality probabilities. Thus, a significant framing effect is evident, which is consistent with previous studies [29]–[31]. The participants regarded the treatment options based on survival rates as opportunities rather than threats. The participants who were provided with mortality information only focused on the threat of death rather than the opportunity for survival. Numerous studies indicate that if decision makers perceive opportunities instead of threats, they become more inclined to take risks; by contrast, if they perceive threats instead of opportunities, they become more conservative [32]–[35]. Therefore, participants exposed to a positive or neutral frame demonstrate a high tendency to take immediate risks for long-term benefits; these risks include selecting the surgery program with a low treatment survival rate but a high five-year survival rate. Another explanation is consistent with CLT. A positive frame describes the treatments in terms of survival rates or reasons in favor (pros) of pursuing the treatment, whereas a negative frame describes the treatments in terms of mortality rates or reasons against (cons) pursuing them. Considering the advantages rather than the disadvantages of high CLs [36], our analysis indicates that decision makers at a high CL perceive desirability rather than feasibility and focus on the long-term goal, compared with those at a low CL. Thus, people exposed to the positive and the neutral frames are more inclined to choose the surgery program than are those exposed to the negative frame.

### 4.3 Interactions between Social Distance and Framing Effects

A significant difference was found in the framing effect at different social distances. However, the framing effect was nonsignificant when the participants decided for themselves, unlike that when participants gave advice to patients. Our findings are inconsistent with those of Marteau to a certain extent [4]. Marteau indicated that the framing effect is significant for doctors at 10% survival rate from surgery but not for patients. The framing effect is nonsignificant for doctors at 40% survival rate but significant for patients. Framing effects are not evident when the survival rate is above 40% for both patients and doctors. In our study, the survival rate from surgery was 50%, and the framing effect was significant for doctors but not for patients. This difference may be attributed to the difference in the number of subjects. The sample number was relatively small in the study by Marteau and the experiment design. Our research materials were not completely the same as those of Marteau. In our study, the responses of the participants to decision-making problems were measured using a 6-point Likert scale, whereas in the study by Marteau, the participants made a dichotomous choice between surgery and radiation therapy. Our results are supported by Thomas, McGreal, and Thielre who reported that the framing of treatment information exerts no influence on the chemical treatment preferred by cancer patients [37].

The influence of social distance on framing effects in the current study is similar to the finding of Nan that framing effects increase when people evaluate for socially distant (e.g., others) versus proximal (e.g., oneself) entities [38]. Further research by Zhong, Shen, and Wu indicates that the framing effect is significant at a long temporal distance but nonsignificant at a short temporal distance [39]. Short-term decision making or independent decision-making results in a low CL, whereas long-term decision making or providing advice to others leads to a high CL. A low CL mostly involves analytical processing, whereas a high CL involves comprehensive processing [8], [40]. Analytical processing primarily involves reassembling dismantled information, relying on actual information rather than the process by which the information is framed. By contrast, comprehensive processing mostly depends on holistic contextual features, making individuals more easily influenced by the framing effect [8]–[10], [40]. Thus, unlike giving advice to others, deciding for oneself is not affected by framing; this finding can be attributed to the different types of processing caused by different CLs.

A significant difference in the effect of social distance was indicated in some frames. Social distance significantly affected medical decisions in the positive and the neutral frames but not in the negative frame. The effect of social distance resulting from the strong influence of the negative frame on decisions was disregarded and attributed to the relatively smaller effects of the positive and the neutral frames on the decision [41]. An alternative explanation can be provided based on regulatory focus theory. Pennington and Roese demonstrated that people’s promotion concerns (i.e., concerns about the positive outcomes of an event) increase as temporal distance increases. By contrast, people’s prevention concerns (i.e., concerns about the negative outcomes of an event) remain constant with temporal distance [42]. Therefore, the salience of prevention concerns is minimally affected by psychological distance.

### 4.4 Conclusion and Implications

The current study demonstrated that both CL and framing depiction significantly affected simulated medical decision making: Compared with giving medical advice for hypothetical patients, people deciding for themselves were far more likely to select conservative radiotherapy. People from positive or neutral frames demonstrated a higher tendency to choose surgery than did those from negative frames. In addition, a significant interaction exists between them.

Therefore, patients being recipients of treatment and vulnerable to medical risk adopt a low CL, whereas doctors as advisers adopt a relatively high CL in selecting a treatment. This variation in CL leads to differences in medical decision making.

In China, the doctor–patient relationship is currently an uncomfortable one [24]. Chinese researchers have often attributed this condition to the imperfect medical system and the decline in doctors’ morality [26]. In Western countries, the attitudes of patients and doctors largely shifted toward patient autonomy during the 1960 s. Prior to this period, doctors made decisions regarding medical treatment without consulting patients or informing them of the options. After the “bioethics revolution” in the 1960 s and the 1970 s, attitudes completely changed; in the West, patient decision making is considered the gold standard. By contrast, no such comprehensive health insurance system exists in China. The doctor- patient relationship in China remains similar to that in Western countries in the 1960 s. Chinese people demonstrate a strong reverence for authority. Doctors are assumed to have relevant knowledge and experience; thus, their decisions are considered more reliable than those of the patients. People often consider decisions as a matter of course. However, the results of the current study suggest that doctors and patients show different tendencies in decision making because of the variation in the CL that they adopt. In addition, doctors seem more easily influenced by framing effects than are patients. 24 Thus, the medical decisions of doctors are not always wiser than those of patients.

The limitations of this study are worth discussing. The major shortcoming is that a simulation instead of an examination of an actual clinical situation was conducted in this study. Whether the decisions made by doctors would match those made by patients in real-life situations is an interesting topic for future research. In addition, a between-subject design involving confounding factors, such as personality and age, was used in the current study. Future studies may explore the moderating roles of personality traits, such as the need for cognition, conscientiousness between CLs, and decision making.
